# Uncovering novel loci for mesocotyl elongation and shoot length in indica rice through genome-wide association mapping

**DOI:** 10.1007/s00425-015-2434-x

**Published:** 2015-11-26

**Authors:** Qing Lu, Mengchen Zhang, Xiaojun Niu, Caihong Wang, Qun Xu, Yue Feng, Shan Wang, Xiaoping Yuan, Hanyong Yu, Yiping Wang, Xinghua Wei

**Affiliations:** State Key Laboratory of Rice Biology, China National Rice Research Institute, Hangzhou, 310006 China

**Keywords:** Direct-seeding, Genome-wide association study, Mesocotyl elongation, Rice (*Oryza sativa* L.), Shoot length

## Abstract

**Totally, 23 loci were detected, and 383 candidate genes were identified, and four of these candidate genes,*Os01g0392100***, ***Os04g0630000***, ***Os01g0904700*and*Os07g0615000*, were regarded as promising targets.**

Direct-seeding cultivation is becoming popular in rice (*Oryza sativa* L.)-planting countries because it is labor- and time-efficient. However, low seedling establishment and slow seedling emergence have restricted the application and popularity of the technique. Mesocotyl elongation and shoot length are two important traits that can enhance rice seedling emergence. A single nucleotide polymorphism (SNP) is a genome sequence variation caused by a single base within a population, and SNPs evenly distributed throughout the genomes of plant species. In this study, a genome-wide association study (GWAS), based on 4136 SNPs, was performed using a compressed mixed linear model that accounted for population structure and relative kinship to detect novel loci for the two traits. Totally, 23 loci were identified, including five loci located known QTLs region. For the mesocotyl elongation, 17 major loci were identified, explaining ~19.31 % of the phenotypic variation. For the shoot length, six major loci were detected, explaining ~39.79 % of the phenotypic variation. In total, 383 candidate genes were included in a 200-kb genomic region (±100 kb of each locus). Additionally, 32 SNPs were identified in 30 candidate genes. Relative expression level analyses indicated that four candidate genes containing SNP variations, *Os01g0392100*, *Os04g0630000*, *Os01g0904700* and *Os07g0615000*, represented promising targets. Finally, eight elite accessions with long mesocotyl and shoot lengths were chosen as breeding donors for further rice direct-seeding variety modifications.

**Electronic supplementary material:**

The online version of this article (doi:10.1007/s00425-015-2434-x) contains supplementary material, which is available to authorized users.

## Introduction

In recent years, as tight labor markets have continued, direct seeding, which is a simple, convenient and resource-efficient cultivation technique that addresses the conflict between rice production and the labor shortage, has become an inevitable trend in rice-growing regions. In particular, deep direct seeding improves not only plant lodging resistance but also rice drought tolerance because roots absorb the deep soil water (Lin et al. 2006). However, poor emergence and weak seedling establishment caused by the deep soil lead to simultaneous yield and grain quality losses, limiting the technique’s application. The mesocotyl and shoot lengths are two crucial agronomic traits for direct seeding because they can enhance seedling establishment and inhibit weed growth (Rao et al. 2007; Lee et al. 2012; Dang et al. 2014).

The mesocotyl is the embryonic tissue located between the coleoptilar node and the basal part of the seedling, and it can directly push the shoot tip above the soil surface during germination (Lee et al. 2012). Therefore, mesocotyl elongation has great significance in rice direct-seeding production. Previous studies have demonstrated that mesocotyl elongation is controlled by multiple genetic factors and environmental signals, such as light (Loercher 1966; Vanderhoef and Briggs 1978), auxin (Cona et al. 2003), abscisic acid (Saab et al. 1992) and jasmonate (Riemann et al. 2003). Other research showed that strigolactones negatively regulate mesocotyl elongation by controlling cell division but not cell elongation in rice during germination and growth in the darkness (Hu et al. 2010). In recent decades, with the rapid development of molecular marker technology, a number of quantitative trait loci (QTLs) for mesocotyl elongation and shoot length were identified using bi-parental linkage mapping in rice. Five mesocotyl elongation and four shoot length QTLs were detected using an F_2_ population by Redoña and Mackill (1996). Eleven QTLs for mesocotyl length were identified using a recombinant inbred line (RIL) population by Cai and Morishima (2002). Additionally, five QTLs related to mesocotyl elongation were mapped on chromosomes 1, 3, 7, 9 and 12 in two independent experiments (Lee et al. 2012).

Although linkage analysis is a useful method for QTLs mapping, the detection of genetic variation is limited because of the doubled parental materials. A genome-wide association study (GWAS), based on the historic recombination in a large natural population, is considered an excellent new strategy to analyze genetic variation and identify valuable genes (or QTLs) for complex traits in genomes. Over the past several years, GWAS was successfully applied in rice (Huang et al. 2010b; Zhao et al. 2011; Spindel et al. 2015), maize (Tian et al. 2011), soybean (Wen et al. 2014; Bao et al. 2015) and wheat (Sukumaran et al. 2015) to uncover genetic variation. In rice, association mapping was used to identify novel loci involved in different complex traits, such as yield (Zhao et al. 2011; Huang et al. 2015; Begum et al. 2015), grain quality (Huang et al. 2010b; Jin et al. 2010), blast resistance (Wang et al. 2014) and environmental stress responses (Cui et al. 2015; Kumar et al. 2015). In addition, 12 trait-marker associations for shoot length were identified using association mapping (Dang et al. 2014). However, until now, there have been limited reports on association mapping for mesocotyl length.

In this study, 469 indica accessions selected from our previous study (unpublished) were used to conduct association mapping. The goals are (1) to analyze genomic variation; (2) to uncover the correlation among direct-seeding emergence, mesocotyl elongation and shoot length; (3) to detect novel loci controlling mesocotyl elongation and shoot length; (4) additionally, to identify elite parental materials for direct-seeding cultivar improvement.

## Materials and methods

### Plant material and genotyping

To reduce spurious associations caused by population structure, a set of 469 global diverse indica accessions with rich genetic diversity (supplementary Table S1), representing the major rice-growing regions, was collected to construct a large association mapping population. All the samples were genotyped using a custom-designed array containing 5291 single nucleotide polymorphisms (SNPs) following the Infinium HD Assay Ultra Protocol (Illumina, Inc. San Diego, CA), with the minor allele frequency <5 % eliminated. Finally, 4136 SNPs were used in our GWAS analyses (supplementary Table S2). These accessions were planted in a randomized complete block design with three replications in a six-column × six-row area in Lingshui (LS; N18°32′, E110°01′) and Hangzhou (HZ; N30°15′, E120°12′) in 2014.

### Mesocotyl elongation and shoot length evaluation

The mesocotyl elongation and shoot length were systematically evaluated in LS and HZ in 2014. Seeds were harvested at 30–40 days after flowering and air-dried under natural conditions. For each accession, 50 plump grains with three replications were treated at 45 °C for 3 days to eliminate dormancy. Then, the grains were wrapped in 20 × 20 cm wet absorbent filter paper, placed vertically, and cultivated at 30 °C in the darkness. During germination, tap water was sprayed to keep the filter paper moist. After 10 days, the mesocotyl and shoot lengths were measured.

### Direct-seeding germination

In total, 24 accessions with long, and 28 with short, mesocotyl lengths were selected to evaluate the difference in seedling emergence at 2 and 5 cm sowing depths and to analyze the effects of different mesocotyl lengths on the speed of emergence (SOE) and final percentage of emergence (POE) in direct-seeding cultivation. For each accession, 50 plump grains were selected manually and soaked in tap water for 2 days, and then pre-germinated at 30 °C for 1 day in the darkness. Germinated seeds were sown in a 12 × 4 cm square, covered with cohesive, continuously moistened fine sand at 2- and 5-cm depths, and then cultivated in a greenhouse at 30 °C. After sowing, the number of emerged seedlings was recorded until the seedling emergence of all accessions remained stable. Then, the mesocotyl and shoot lengths were measured after being carefully cleaned. The experiment was performed with two replications.

### Data analysis

The mean, standard error (SE) and broad-sense heritability ($H_{B}^{2}$) were calculated using Excel 2007. The mean and SE were computed by AVERAGE () and STDEV/SQRT (COUNT ()), respectively. The $H_{B}^{2}$ was computed by an ANOVA using the following formula:

$$H_{B}^{2} \, = \, \sigma_{g}^{2} / \, \left( {\sigma_{g}^{2} + \sigma_{e}^{2} } \right),$$

where $\sigma_{g}^{2}$ is genetic variance and $\sigma_{e}^{2}$ is error variance. The interactions of genotype × environment (G × E) were analyzed using an ANOVA in SAS system 9.0 (SAS, Inc., Cary, NC, USA), as was the percentage of phenotypic variation explained by population structure ($R_{\text{PCA}}^{2}$ %). Correlation coefficients were computed using R “corrgram” package (https://cran.r-project.org/web/packages/corrgram/), and the best linear unbiased prediction (BLUP) was carried out in R “lme4” package (https://cran.r-project.org/web/packages/lme4/) for the estimation of mesocotyl and shoot length phenotypic values in two environments.

### Population structure, kinship and association mapping

The population structure was evaluated by Structure version 2.2 with a Bayesian Markov Chain Monte Carlo model (Pritchard et al. 2000), and each number of populations was set from 1 to 15 with five runs, 10,000 burn-in period and 100,000 Markov Chain Monte Carlo replications (Evanno et al. 2005). A principal component analysis (PCA) was also used to verify the population structure using PowerMarker version 3.25 (Liu and Muse 2005) and NTSYSpc version 2.1 (Rohlf 2000). Population differentiation statistics (*F*_ST_) among different subpopulations were calculated using PowerMarker version 3.25 (Liu and Muse 2005). The pairwise relatedness coefficients were calculated under SPAGeDi version 1.4c, and all of the negative values in the results were set as zero (Hardy and Vekemans 2002). Linkage disequilibrium (LD) parameter (*r*^2^) for estimating the degree of LD between pairwise SNPs was calculated under TASSEL version 4.0 (Bradbury et al. 2007), and the LD decay rate was the chromosomal distance at which the average pairwise correlation coefficient dropped to half its maximum value (Huang et al. 2010b).

A GWAS was performed in TASSEL version 4.0 (Bradbury et al. 2007), and the EMMA (Kang et al. 2008) and P3D (Zhang et al. 2010) algorithms were used to reduce computing time. The compressed mixed linear model (cMLM) with the top 10 PCs and the relative kinship matrix (*K*) as covariates was used to reduce false-positive associations (Price et al. 2006). As Bonferroni correction (1/4136 = 2.4E-04) was too conservation (Nakagawa 2004), a compromised threshold of *P* = 0.001 was used to screen the significant associations. According to whole genome average LD decay distances in our previous study (unpublished), the candidate genes were predicted within a 200-kb genomic region (±100 kb of each significant locus). The genes and annotations were obtained from the Rice Haplotype Map Project Database (http://202.127.18.221/RiceHap2/).

### RNA extraction and qRT-PCR

The expression levels of mesocotyl and shoot length candidate genes were analyzed by quantitative real-time PCR (qRT-PCR) using two groups of accessions with completely different phenotypes, and each group contained three typical accessions. For each accession, 50 plump grains were germinated at 30 °C for 10 days in the darkness. Total RNA was extracted from the mesocotyl and shoot tissues using a MiniBEST Plant RNA Extraction kit (Takara Bio Inc, Japan). First-strand complementary DNA (cDNA) was synthesized by PrimeScript RT Master Mix (Takara Bio Inc, Japan). The reaction mixture was run on an Applied Biosystems 7500 Real-Time PCR system (Applied Biosystems, Carlsbad, CA, USA). Three replicates were performed for each sample. Rice *Ubq*-*2* was used as the internal control in all analyses. The primers for qRT-PCR are listed in supplementary Table S3.

## Results

### Material distribution and phenotypic variation

**Fig. 1:**
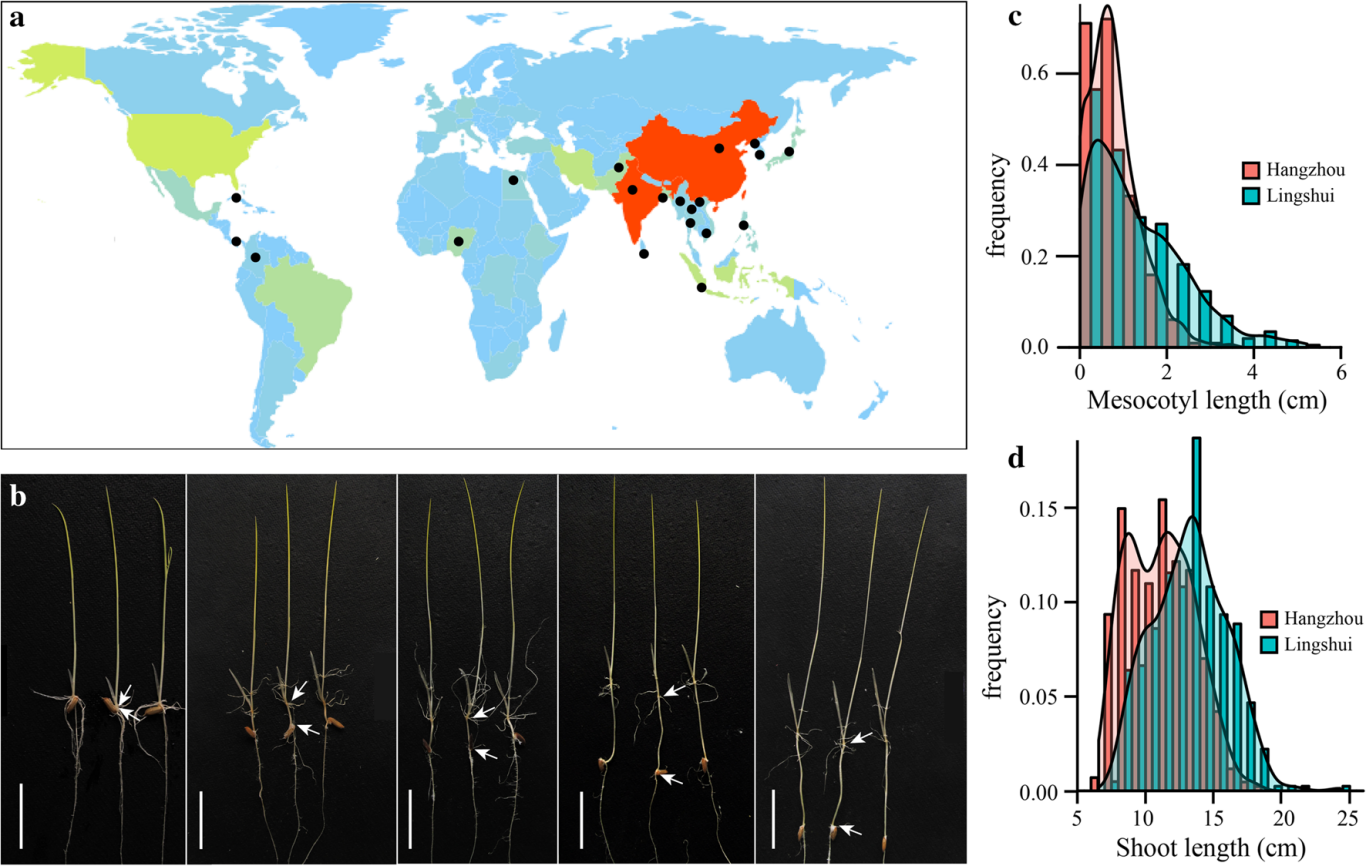
Material distribution and mesocotyl elongation diversity. **a** The worldwide distribution of 469 indica accessions. The *black dots* represent the country-specific distribution. **b** Different mesocotyl lengths in the germplasm. *Bar* 3 cm. **c** Histogram of mesocotyl lengths in Hangzhou and Lingshui. **d** Histogram of shoot lengths in Hangzhou and Lingshui

In our study, 469 indica accessions collected from 20 countries (Fig. 1a) were used as the GWAS panel (supplementary Table S1). The germplasm resources maintain rich mesocotyl elongation and shoot length variations (Fig. 1b–d), and the phenotypic variations are summarized in Table 1 and supplementary Table S4. The G × E interactions were highly significant (*P* < 0.001) across the two environments, indicating that environment effect should not be ignored. For the mesocotyl length, the phenotypic variation explained by the population structure ($R_{\text{PCA}}^{2}$) was 31.33 and 9.21 % in the two environments, respectively, and the broad-sense heritability ($H_{B}^{2}$) was 61.83 %. For the shoot length, the $R_{\text{PCA}}^{2}$ was 50.20 and 45.42 % across the two environments, and the $H_{B}^{2}$ reached 77.32 %. Thus, the results suggested that the effects of the population structure and environment should be taken into consideration in further GWAS analyses.

**Table 1:** Phenotypic variation for mesocotyl and shoot lengths across two environments in 469 indica accessions

Trait	Environment	Mean (cm) ± SE	Range (cm)	$R_{\text{PCA}}^{2}$ (%)^a^	$H_{B}^{2}$ (%)^b^	G × E^c^
ML	LS, 2014	1.28 ± 0.05	0.00–5.22	31.33	61.83	***
	HZ, 2014	0.77 ± 0.03	0.00–3.45	9.21		
SL	LS, 2014	13.24 ± 0.12	7.63–24.28	50.20	77.32	***
	HZ, 2014	11.10 ± 0.11	6.56–18.12	45.42		

*ML* Mesocotyl length, *SL* Shoot length, *LS* Lingshui, *HZ* Hangzhou, *SE*, standard error

^a^Phenotypic variation explained by population structure

^b^Broad-sense heritability

^c^G × E, interaction of genotype and environment

*** Significant at *P* = 0.001

### Analysis of direct-seeding germination

**Fig. 2:**
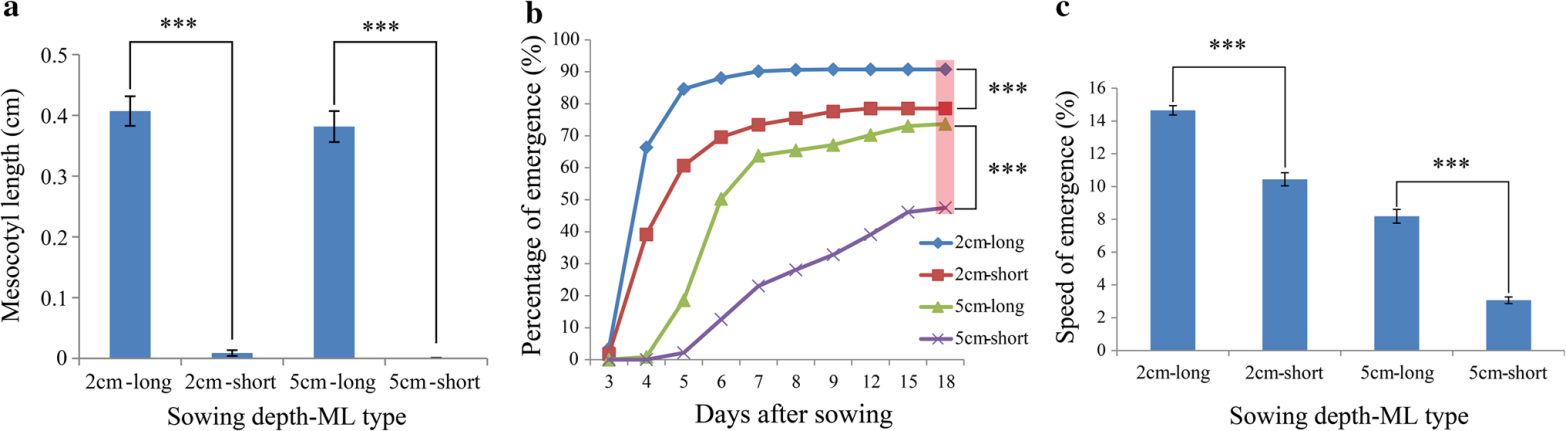
Direct-seeding emergence of long and short mesocotyl length accessions at two sowing depths. **a** Two groups of mesocotyl lengths in two sowing depths. *Error bar* standard error. **b** Cumulative percentage of emergence in 18 days. The *pink area* represents Duncan’s multiple comparison on the 18th day. **c** Speed of emergence at two sowing depths. *Asterisks* Significant at 0.001. *Error bar* standard error. *ML-type* Mesocotyl length type

In total, 52 accessions, which were divided into two groups, one with long and the other with short mesocotyl lengths, were selected to analyze the POE and SOE. SOE was the average POE per day before the germination rate stopped significantly increasing. The sowing depths of 2 and 5 cm represent shallow overburden and deep seeding, respectively. The results are summarized in Fig. 2 (supplementary Figure S1; supplementary Table S5). The mesocotyl lengths between the two groups were highly different independent of the sowing depth (Fig. 2a). For the 2-cm sowing depth, the POE of the long mesocotyl accessions was significantly greater than that of the short mesocotyl accessions, reaching 90.76 % (Fig. 2b). In addition, the POEs of long and short mesocotyl groups remained stable (not significant at the 0.05 level) after the sixth and seventh days, respectively (supplementary Table S5). For the 5-cm sowing depth, the POE of the short mesocotyl accessions was the lowest, only up to 47.49 % (Fig. 2b). Moreover, until the 15th day, the emergence rate was unchanged and occurred 7 days after that of the long mesocotyl accessions (supplementary Table S5). For the SOE trait, regardless of the sowing depth, the long mesocotyl group had a higher emergence rate than that of the short one (Fig. 2c). Based on the results, we speculated that the POE and SOE traits may be related to the mesocotyl length.

**Fig. 3:**
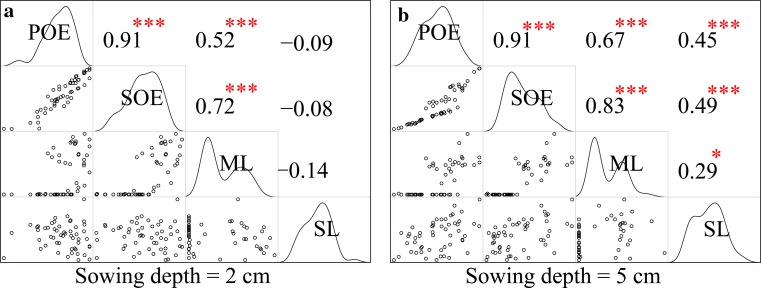
Correlation of four direct-seeding traits at two sowing depths. The *upper panel* contains the correlation coefficients, and the *lower panel* contains the distributions of the two traits. The *diagonal* represents the density line of the traits. *Single asterisk* and *Triple asterisk* represent significant at 0.05 and 0.001, respectively. *POE* percentage of emergence, *SOE* speed of emergence, *ML* mesocotyl length and *SL* Shoot length

Additionally, the correlations among POE, SOE, mesocotyl length and shoot length were analyzed, and the results showed that POE and SOE were highly positively correlated with mesocotyl length at 2- and 5-cm sowing depths (Fig. 3a, b). Moreover, in the deep soil condition, the POE and SOE were also positively correlated with shoot length, but the correlation coefficients were lower than those of the mesocotyl length (Fig. 3b). These results indicated that mesocotyl length is a very important agronomic trait in direct seeding, especially in deep direct seeding. Uncovering novel loci for mesocotyl and shoot lengths has a great significance for direct-seeding rice variety breeding in the future.

### Population structure, kinship and linkage disequilibrium estimation

**Fig. 4:**
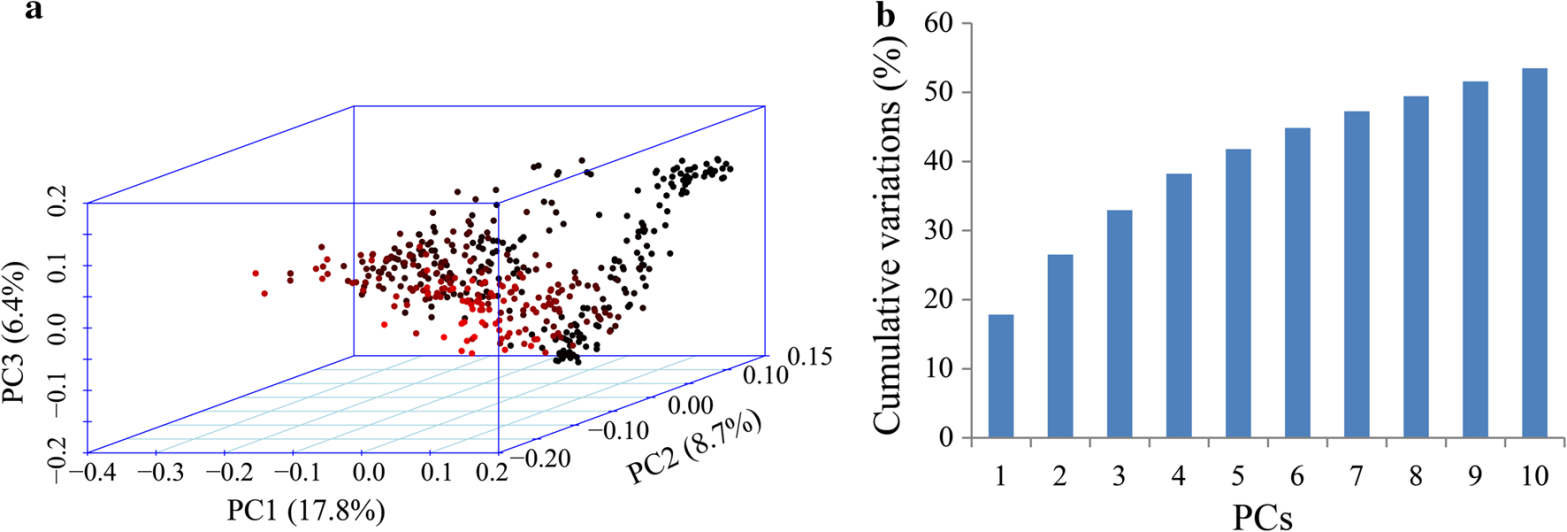
Population structure of 469 indica accessions. **a** *Different colors* represent different ingredients, and PC1, PC2 and PC3 are the top three PCs. **b** Cumulative variations explained by the top 10 PCs

Our previous work (unpublished) indicated that the indica panel can be classed into four subpopulations (supplementary Figure S2) with moderate differentiation (*F*_*ST*_ values ranged from 0.05 to 0.25), which was also supported by the neighbor-joining tree (supplementary Figure S3). The PCA results showed that PC1, PC2 and PC3 accounted for 17.8, 8.7 and 6.4 % of the genetic variation, respectively (Fig. 4a). Moreover, the top 10 PCs explained ~53.5 % of the total variation in population structure (Fig. 4b), and these were used as covariates in the next GWAS analyses (supplementary Table S6). The kinship analysis suggested that there was no or weak relatedness (83.0 % pairwise kinship coefficients ≤0.1) in the GWAS panel. In addition, the genome-wide LD decay distance was ~109.37 kb.

### Association mapping for mesocotyl and shoot lengths

A number of significant trait-marker associations were detected in our study. To select the major associations, the loci with the lowest *P* values were maintained in a 200-kb genome region (Wang et al. 2014). After clumping, total of 23 significant loci were obtained using the BLUP phenotypic values (Table 2).

**Table 2:** Summary of SNPs significantly associated with mesocotyl and shoot lengths using the BLUP method

Trait	Marker	Chr.	Position	Allele^a^	*P* value	*R* ^2^ (%)^b^	Known locus
ML	seq-rs9	1	328,402	A/G	1.31E-04	19.31	
	seq-rs69	1	2,310,793	C/T	7.28E-04		
	seq-rs303	1	16,819,798	C/T	9.65E-04		
	seq-rs576	1	37,645,391	G/A	5.12E-04		*qMel*-*1* (Lee et al. 2012)
	seq-rs2224	4	26,977,890	A/G	2.69E-04		
	seq-rs2230	4	27,764,966	T/C	1.32E-04		
	seq-rs2327	4	32,369,445	C/T	5.52E-04		
	seq-rs2346	4	32,780,816	T/C	5.51E-04		
	seq-rs2638	5	27,312,765	C/A	6.16E-04		
	seq-rs2709	6	1,587,323	T/C	8.34E-04		*qML6* (Huang et al. 2010a)
							SNP No: 0615693545 (Wu et al. 2015)
	seq-rs3113	6	30,282,802	G/A	4.57E-04		
	seq-rs3130	6	31,389,107	T/C	8.99E-04		
	seq-rs3224	7	6,123,504	A/G	2.25E-04		
	seq-rs4080	9	1,597,258	A/G	1.00E-03		
	seq-rs4094	9	4,209,107	T/C	1.00E-03		
	seq-rs4192	9	10,045,572	A/G	1.69E-04		*qMel*-*9* (Lee et al. 2012)
	seq-rs5352	11	26,779,678	A/G	8.12E-04		
SL	seq-rs609	1	41,143,657	G/A	1.25E-05	39.79	RM5398 (Dang et al. 2014)
	seq-rs3537	7	25,936,314	G/A	1.16E-03		RM234 (Dang et al. 2014)
	seq-rs3541	7	26,021,870	G/A	1.56E-04		RM234 (Dang et al. 2014)
	seq-rs3546	7	26,498,542	G/A	1.34E-03		RM234 (Dang et al. 2014)
	seq-rs3663	8	1,092,192	G/A	1.21E-03		
	seq-rs4250	9	14,781,791	A/C	1.24E-03		

*ML* mesocotyl length, *SL* Shoot length

^a^Major allele/minor allele

^b^Phenotypic variation explained by all of the significant loci

**Fig. 5:**
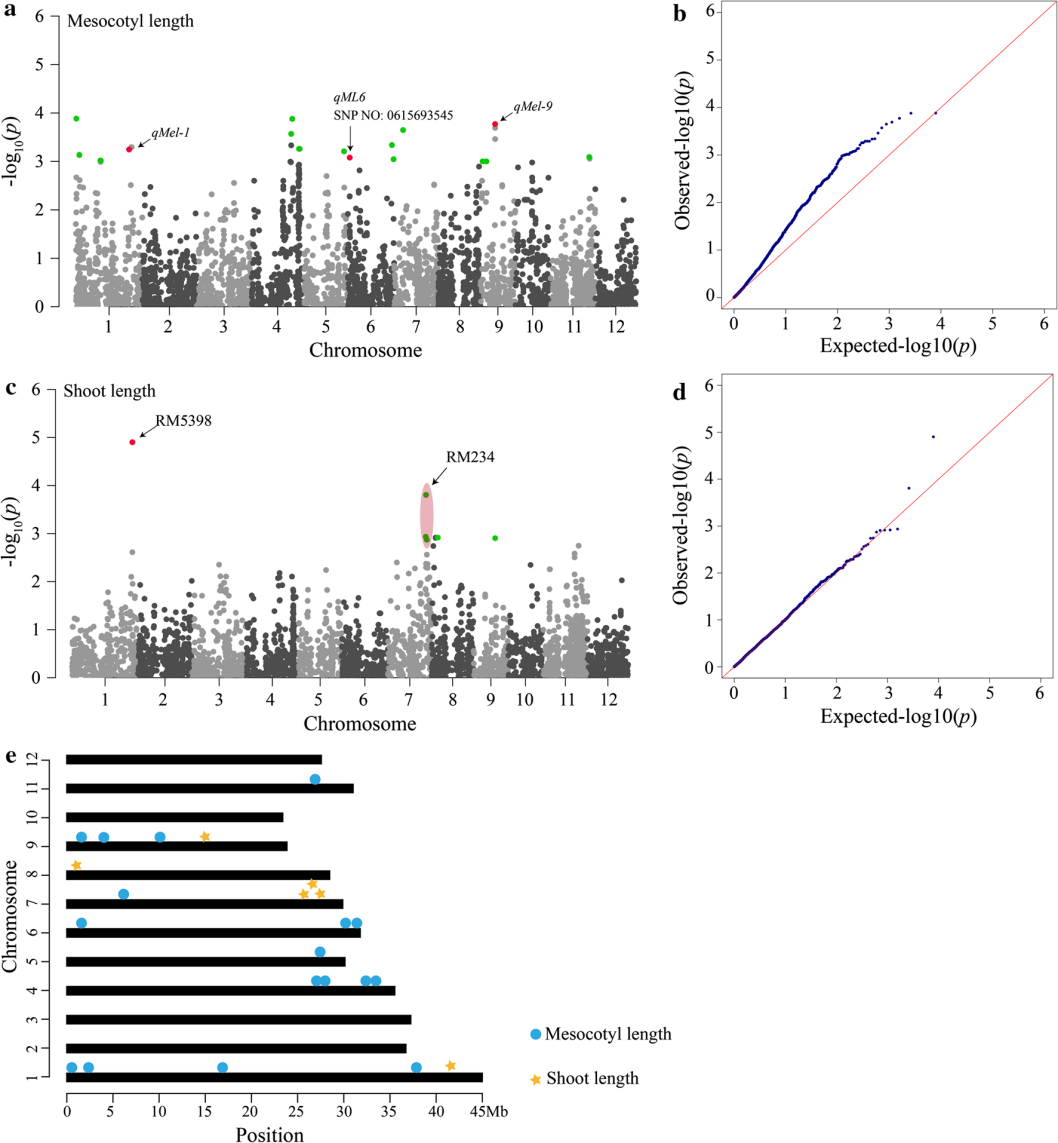
Manhattan plots, quantile–quantile plots and the distribution of significant GWAS loci. **a** and **c** Manhattan plots showing *P* values along the whole genome. *Green dots* represent new significant loci, and *red dots* represent known QTLs. **b** and **d** Quantile–quantile plots showing the expected null distribution of *P* values (expected *P* values), assuming no association, represented as a *red line* and distribution of *P* values (observed *P* values) represented as *blue dots*. **e** The distribution of 23 loci on 12 chromosomes. *Blue dots* and *yellow stars* represent mesocotyl and shoot length associations, respectively

For the mesocotyl length, 17 major loci, explaining ~19.31 % of the phenotypic variation, were identified on chromosomes 1, 4, 5, 6, 7, 9 and 11, and three loci were close to locations identified in previous reports (Huang et al. 2010a; Lee et al. 2012; Wu et al. 2015) (Table 2; Fig. 5a, e). Moreover, six and seven significant loci were detected in LS and HZ environments, respectively (supplementary Table S7). The loci, seq-rs2703 and seq-rs2709, on chromosome 6 were identified in both environments (supplementary Figure S4a, c; supplementary Table S7). Three loci (seq-rs9, seq-rs2224 and seq-rs2327) were detected in LS environment and using the BLUP method, and one locus (seq-rs2709) was obtained in HZ environment and using the BLUP method (supplementary Figure S5a).

For the shoot length, six major loci, explaining ~39.79 % of the phenotypic variation, were detected on chromosomes 1, 7, 8 and 9, and four loci overlapped with previously identified trait-marker associations (Dang et al. 2014) (Table 2; Fig. 5c, e). In addition, two and one significant trait-marker associations were identified in LS and HZ environments, respectively (supplementary Table S7). Additionally, the locus, seq-rs609, which was also detected by Dang et al. (2014) using GWAS strategy, was obtained across two environments and using the BLUP method in our association mapping (supplementary Figure S4e, g; supplementary Figure S5b).

### Candidate gene analysis

**Fig. 6:**
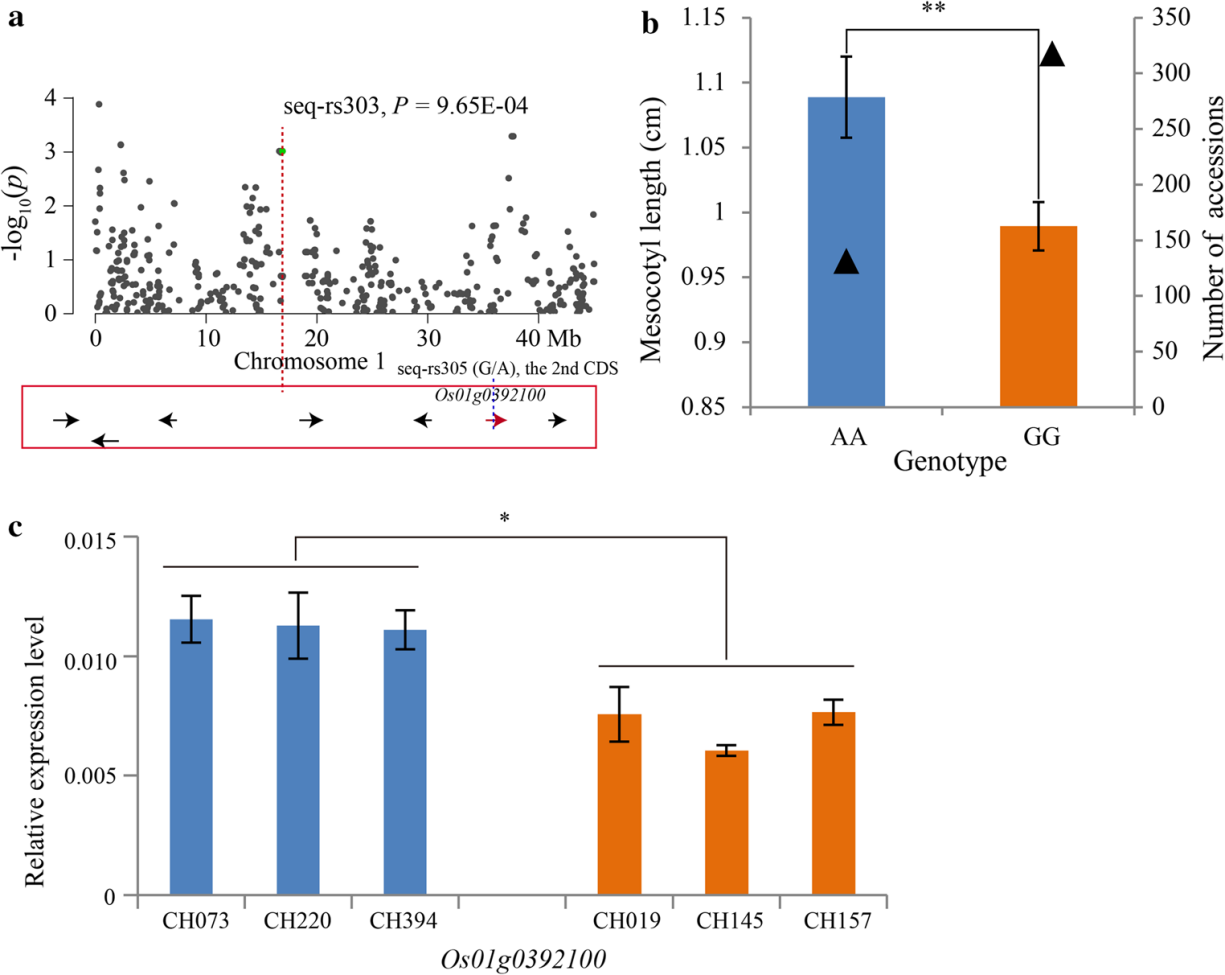
Summary of the mesocotyl length candidate gene, *Os01g0392100*, resulting from GWAS. **a** Association peak of *Os01g0392100* with a SNP located in the second CDS region. All of the arrows represent candidate genes within a 200-kb region; *red arrow* represents the candidate gene with a SNP variation showed by *blue dotted line*. **b** Mesocotyl lengths of the two genotypes at the seq-rs305 (G/A). The *triangles* represent the number of accessions. *Error bar* standard error. **c** The relative expression levels of *Os01g0392100* in long (CH073, CH220 and CH394) and short (CH019, CH145 and CH157) mesocotyl length accessions. *Error bar* standard deviation. *Single asterisk* and *Double asterisk* represent *t* test at 0.05 and 0.01 significance level, respectively

**Fig. 7:**
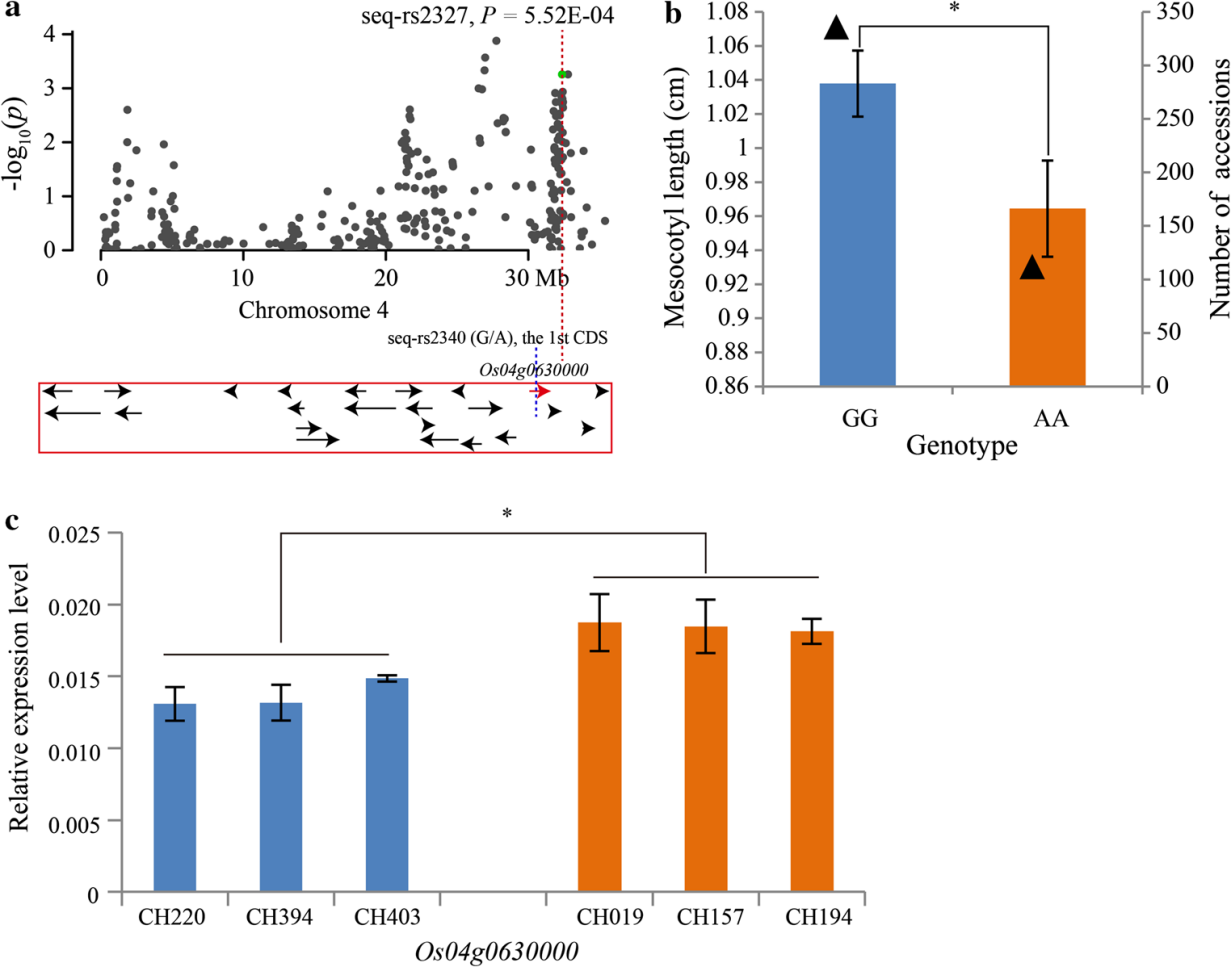
Summary of the mesocotyl length candidate gene, *Os04g0630000*, resulting from GWAS. **a** Association peak of *Os04g0630000* with a SNP located in the first CDS region. All of the *arrows* represent candidate genes within a 200-kb region; *red arrow* represents the candidate gene with a SNP variation showed by *blue dotted line*. **b** Mesocotyl lengths of the two genotypes at the seq-rs2340 (G/A). The *triangles* represent the number of accessions. *Error bar* standard error. **c** The relative expression levels of *Os04g0630000* in long (CH220, CH394 and CH403) and short (CH019, CH157 and CH194) mesocotyl length accessions. *Error bar* standard deviation. *Asterisk* represents *t* test at 0.05 significance level, respectively

**Fig. 8:**
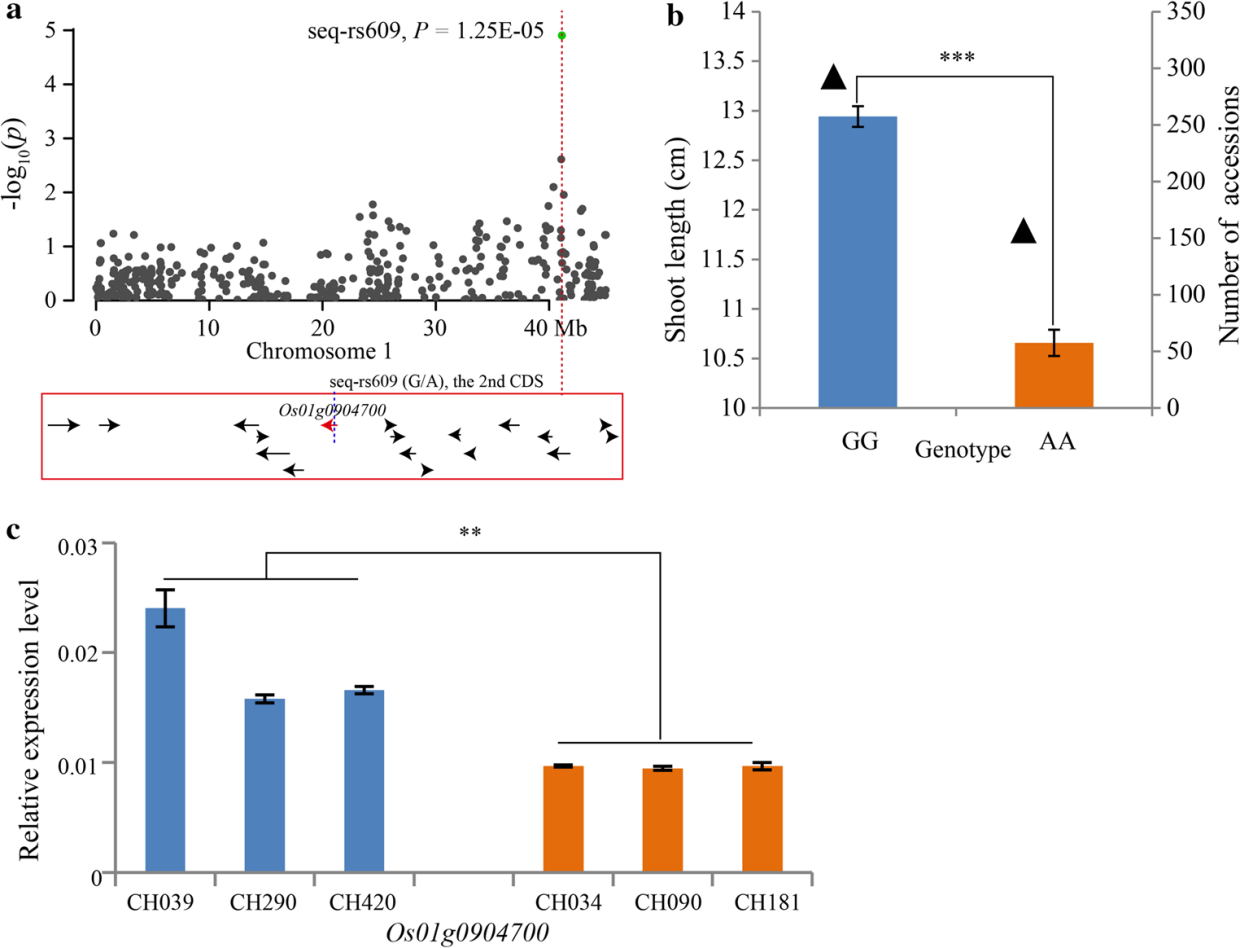
Summary of the shoot length candidate gene, *Os01g0904700*, resulting from GWAS. **a** Association peak of *Os01g0904700* with a SNP located in the second CDS region. All of the *arrows* represent candidate genes within a 200-kb region; *red arrow* represents the candidate gene with a SNP variation showed by *blue dotted line*. **b** Shoot lengths of the two genotypes at the seq-rs609 (G/A). The *triangles* represent the number of accessions. *Error bar* standard error. **c** The relative expression levels of *Os01g0904700* in long (CH039, CH290 and CH420) and short (CH034, CH090 and CH181) shoot length accessions. *Error bar* standard deviation. *Double asterisk* and *triple asterisk* represent *t* test at 0.01 and 0.001 significance level, respectively

**Fig. 9:**
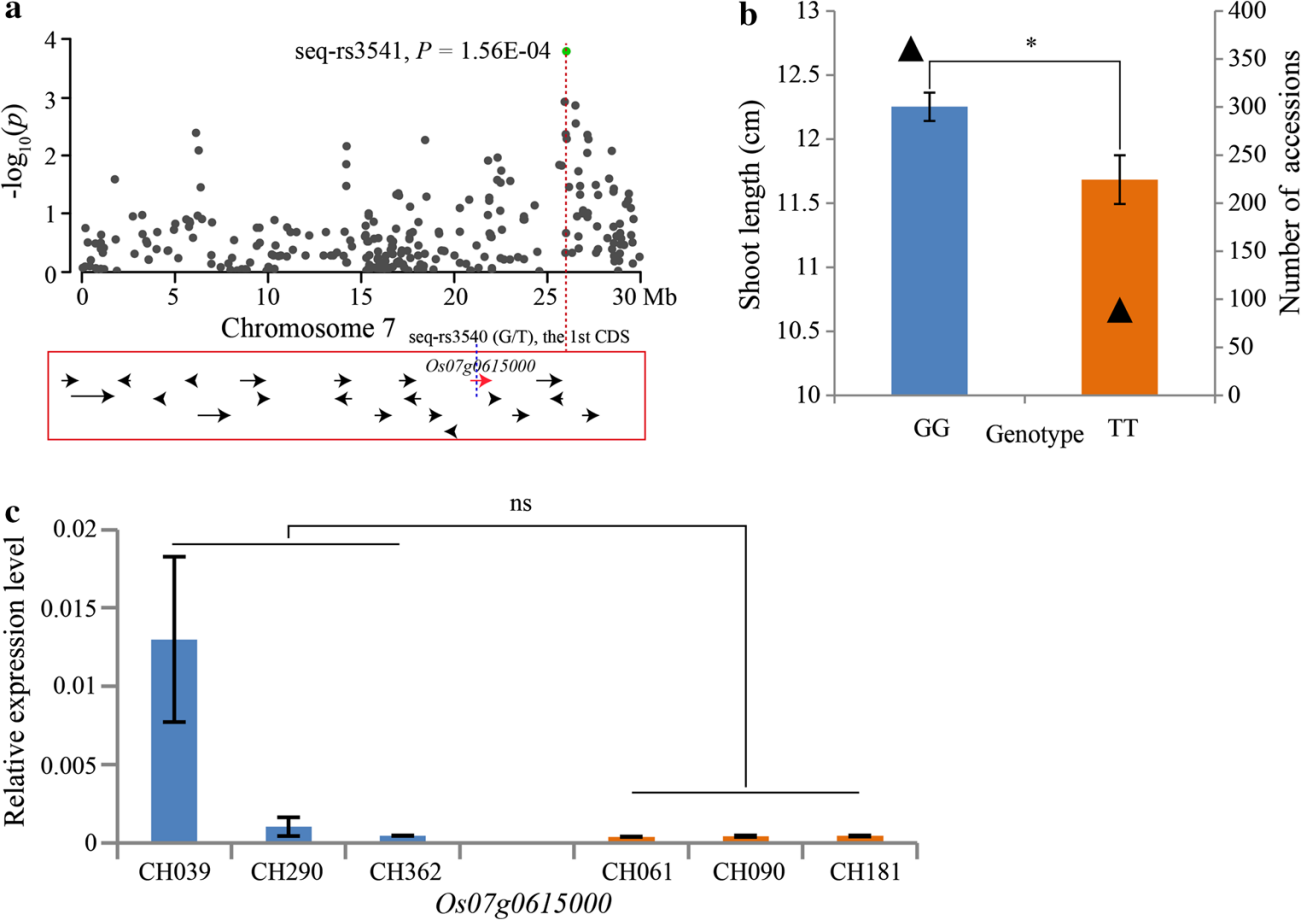
Summary of the shoot length candidate gene, *Os07g0615000*, resulting from GWAS. **a** Association peak of *Os07g0615000* with a SNP located in the first CDS region. All of the *arrows* represent candidate genes within a 200-kb region; *red arrow* represents the candidate gene with a SNP variation showed by *blue dotted line*. **b** Shoot lengths of the two genotypes at the seq-rs3540 (G/T). The *triangles* represent the number of accessions. *Error bar* standard error. **c** The relative expression levels of *Os07g0615000* in long (CH039, CH290 and CH362) and short (CH61, CH090 and CH181) shoot length accessions. *Error bar* standard deviation. *Asterisk* represents *t* test at 0.05 significance level, and ns represents not significant at 0.05 level

For the 23 significant loci, 383 candidate genes were obtained within a 200-kb genomic region (±100 kb from the peak SNP) from the Rice Haplotype Map Project Database (http://202.127.18.221/RiceHap2/) (supplementary Table S8). In addition, the distributions of the 4136 SNPs were analyzed and 32 SNPs were located in 30 candidate genes for the two traits (supplementary Table S9). Moreover, the average phenotypic values of accessions carrying each SNP allele were examined. Interestingly, the phenotypic values of the two genotypes of the 13 different candidate genes reached significant (*P* = 0.05) or highly significant (*P* = 0.01 or 0.001) levels (Supplementary Table S9). We speculated that the phenotypic variation may be caused by these SNP changes. More importantly, four SNPs were discovered to be located in the coding sequence (CDS) region in four of the 13 different candidate genes. For example, *Os01g0392100*, encoding a conserved hypothetical protein, was located near seq-rs303 (*P* = 9.65E-04) that was significantly associated with the mesocotyl length, and SNP seq-rs305 (G/A) was located in the second CDS region (Fig. 6a). The phenotypic value of the AA genotype was significantly greater than that of GG (Fig. 6b). The expression levels in the long mesocotyl length accessions were higher than that in the short ones (Fig. 6c; supplementary Table S10). *Os04g0630000*, encoding a SDA1 domain-containing protein, was detected in SNP seq-rs2327 (*P* = 5.52E-04), which was significantly related to the mesocotyl length, and SNP seq-rs2340 (G/A) was located in the first CDS region (Fig. 7a). The phenotypic value of the GG genotype was significantly greater than that of AA (Fig. 7b). On the contrary, *Os04g0630000* showed a lower relative expression level in the long group (Fig. 7c; supplementary Table S10). The result implied that the candidate gene may be a negative regulator of the mesocotyl length. *Os01g0904700*, similar to the two-component response regulator ARR1, was located near seq-rs609 (*P* = 1.25E-05), which was a shoot length QTL. The SNP seq-rs609 (G/A) was located in the second CDS region (Fig. 8a), and the phenotypic value of the GG genotype was significantly greater than that of AA (Fig. 8b). Further qRT-PCR analyses revealed that the expression levels of the *Os01g0904700* increased dramatically in the long shoot length group (Fig. 8c; supplementary Table S10). *Os07g0615000*, encoding a pentatricopeptide repeat-containing protein, was predicted near seq-rs3541 (*P* = 1.56E-04), which correlated with the shoot length. The SNP, seq-rs3540 (G/T), was observed in the first CDS region (Fig. 8a), and the phenotypic value of the GG genotype was significantly greater than that of AA (Fig. 8b). Although the average expression level was not significant between the two groups, two accessions, CH039 (Shouguzao) and CH290 (Zaobai), with long mesocotyl lengths displayed a higher expression level (Fig. 9c). Moreover, the average fold change was the highest, up to ~10.98, among the four candidate genes (supplementary Table S10). Thus, all of the four candidate genes can be regarded as the most promising positive or negative regulators of mesocotyl elongation and shoot length. In addition, eight accessions with long mesocotyl and shoot lengths across two environments were obtained to modify rice direct-seeding varieties in the future (supplementary Table S11).

## Discussions

The direct seeding of rice is an important development because it is simple, convenient and labor- and time-efficient. However, low seedling establishment and slow seedling emergence rates have limited the application and popularity of the direct-seeding technique. The mesocotyl, as a bridge that connects the basal part of the seedling with the coleoptilar node and shoot, pushes the shoot tip above the soil surface during germination. Previous work showed that the elongation of the mesocotyl served to elevate the coleoptile and shoot to allow the primary leaves to emerge (Mgonja et al. 1994) and demonstrated that mesocotyl length had a great influence on seedling establishment in rice (Luo et al. 2007). Our results showed that the long mesocotyl accessions had greater seedling establishment and higher emergence rates independent of the 2 or 5-cm sowing depths (Fig. 2b, c). An analysis of the correlations of direct-seeding traits showed that POE and SOE were highly positively correlated with mesocotyl length at 2- and 5-cm sowing depths. Additionally, in deep soil conditions, the POE and SOE were also positively related to shoot length (Fig. 3). These results indicated that mesocotyl length is a vital factor in deep direct seeding. Moreover, previous research showed that a longer length of the mesocotyl in upland rice might be an adaptation that boosts seedling emergence from deep soils (Wu et al. 2005). To date, limited QTLs related to mesocotyl and shoot lengths have been identified. Therefore, detecting novel loci is necessary for rice breeding in the future.

Mesocotyl elongation is vulnerable to environmental influences, such as light (Loercher 1966; Vanderhoef and Briggs 1978), moisture (Takahashi 1978), plant hormones (Saab et al. 1992; Cona et al. 2003; Riemann et al. 2003) and sowing depth (Alibu et al. 2012). Here, the G × E interaction was highly significant (*P* < 0.001) (Table 1). This may be due to the environmental effects, for example differences in moisture or temperature during experiments. To reduce the false-positive associations caused by environment, the BLUP model was used to predict the phenotypic values. The method was also performed in the GWAS of soybean and maize (Xue et al. 2013; Wen et al. 2014). Moreover, in our GWAS panel, the phenotypic variation explained by population structure ranged from 9.21 to 50.20 % for the two traits (Table 1), and a weak relative relatedness also existed in our previous analyses (unpublished). This suggested that population structure and relative kinship were other factors that increased the type I error rate, and thus the cMLM model with the top 10 PCs and kinship matrix as covariates was used to adjust the GWAS results (Price et al. 2006; Zhang et al. 2010).

Previous reports showed that some QTLs for mesocotyl elongation and shoot length were identified using linkage analysis or association mapping in the past few years (Redoña and Mackill 1996; Cai and Morishima 2002; Lee et al. 2012; Dang et al. 2014). Interestingly, the QTLs for mesocotyl elongation mainly concentrated on chromosomes 1 and 3 in different mapping populations and experimental conditions, such as *qMel*-*1* and *qMel*-*3*, which were fine mapped to 3799- and 6964-kb intervals, respectively (Lee et al. 2012). Additionally, on chromosome 6, *qML6* for mesocotyl length, which was detected by Huang et al. (2010a) and Wu et al. (2015), was also identified in our GWAS study under two environments (supplementary Table S7; supplementary Figure S4a, c) and using the BLUP method (Table 2). Based on the genome LD decay, 22 candidate genes were predicted from the Rice Haplotype Map Project Database (seq-rs2709) (supplementary Table S8). Moreover, a mesocotyl length QTL, *qMel*-*12*, which was mapped on chromosome 12 by Lee et al. (2012), was also detected in our study under HZ environment (supplementary Table S7). In addition, two QTLs, *qMel*-*1*and *qMel*-*9* (Lee et al. 2012) for mesocotyl length, were also identified using the BLUP method (Table 2). Based on the whole genome LD decay, we finally restricted the two loci, which was associated with the seq-rs576 (*P* = 5.12E-04) and the seq-rs4192 (*P* = 1.69E-04), to a 200-kb genomic region, and 25 and 17 candidate genes were obtained, respectively (supplementary Table S8). However, on chromosomes 2, 3, 8 and 10, there was no significant association for mesocotyl length, possibly due to the QTL effects being too small or the marker density being too low to detect any QTLs. For the shoot length, the seq-rs609 locus, which was identified in previous study (Dang et al. 2014), was detected under the two environments (supplementary Table S7; supplementary Figure S4e, g) and using the BLUP method (Table 2), and 19 candidate genes were obtained (supplementary Table S8). Three loci located near the RM234 marker on chromosome 7 (Dang et al. 2014) were also detected (Table 2). These results indicated that the QTLs controlling mesocotyl elongation and shoot length existed on chromosomes 1, 4, 5, 6, 7, 8, 9 and 11.

In our study, 23 major loci (Table 2) containing 383 candidate genes (supplementary Table S8) were obtained. Further analyses revealed that 32 SNPs were located in 30 candidate genes (supplementary Table S9) and four were located in CDS regions in different candidate genes having significant phenotypic variation (Figs. 6a, b; 7a, b; 8a, b; 9a, b). The qRT-PCR analyses indicated that the expression levels of three candidate genes had a significant difference between the long and short groups (Figs. 6c; 7c; 8c). Although the expression level of *Os07g0615000* was not significant between the two groups, two accessions, CH039 (Shouguzao) and CH290 (Zaobai), showed a higher expression level (Fig. 9c), and the average fold change of long group reached to ~10.98 (supplementary Table S10). Consequently, we speculated that the four genes may be the most promising choices for the regulation of mesocotyl elongation and shoot length. Despite the relative expression verification, all of the candidate genes need to be validated in future using biotechnology methods, such as genetic transformation and transferred DNA insertion.

In this study, association mapping, with corrections for genetic structure and relative kinship, was used to identify multiple novel loci correlated with mesocotyl elongation and shoot length, and known QTLs were refined to a narrower region. Moreover, new candidate genes were obtained, and genetic variations combined with phenotypic variances were observed. These results indicated that GWAS can be used as a powerful strategy for uncovering new loci in complex traits and provides rich genetic variation information and valuable markers for rice breeding. In addition, these candidate genes containing SNP variations represent promising targets for further validation efforts. The validated accessions with excellent phenotypes will be used as breeding parents for direct-seeding rice cultivar improvement in the future.

### *Author contribution statement*

XHW designed the experiments. QL, MCZ, XJN and SW performed the phenotyping. QL, QX and YF performed the SNP genotyping. XPY, HYY and YPW managed the materials. QL analyzed the data and drafted the manuscript. CHW helped to revise the manuscript. All of the authors read and approved the final manuscript.

## Electronic supplementary material

Supplementary material 1 (PDF 432 kb)

Supplementary material 2 (PDF 44 kb)

Supplementary material 3 (PDF 122 kb)

Supplementary material 4 (PDF 309 kb)

Supplementary material 5 (PDF 75 kb)

Supplementary material 6 (XLSX 27 kb)

Supplementary material 7 (XLSX 150 kb)

Supplementary material 8 (XLSX 15 kb)

Supplementary material 9 (XLSX 34 kb)

Supplementary material 10 (XLSX 15 kb)

Supplementary material 11 (XLSX 74 kb)

Supplementary material 12 (XLSX 15 kb)

Supplementary material 13 (XLSX 30 kb)

Supplementary material 14 (XLSX 17 kb)

Supplementary material 15 (XLSX 18 kb)

Supplementary material 16 (XLSX 358 kb)

## Abbreviations

ANOVA: Analysis of variance
BLUP: Best linear unbiased prediction
cMLM: Compressed mixed linear model
G × E: Interaction of genotype and environment
GWAS: Genome-wide association study
LD: Linkage disequilibrium
ML: Mesocotyl length
PCA: Principle component analysis
POE: Percentage of emergence
QTL: Quantitative trait locus
SE: Standard error
SL: Shoot length
SNP: Single nucleotide polymorphism
SOE: Speed of emergence
