# Supplementary material for: Uncovering novel loci for mesocotyl elongation and shoot length in indica rice through genome-wide association mapping
Source: Planta. 2015 Nov 26;243:645–57. doi: 10.1007/s00425-015-2434-x (PMC4757631; doi:10.1007/s00425-015-2434-x)
Supplement: Supplementary file 1 — Supplementary material 1 (PDF 432 kb) [file 425_2015_2434_MOESM1_ESM.pdf]

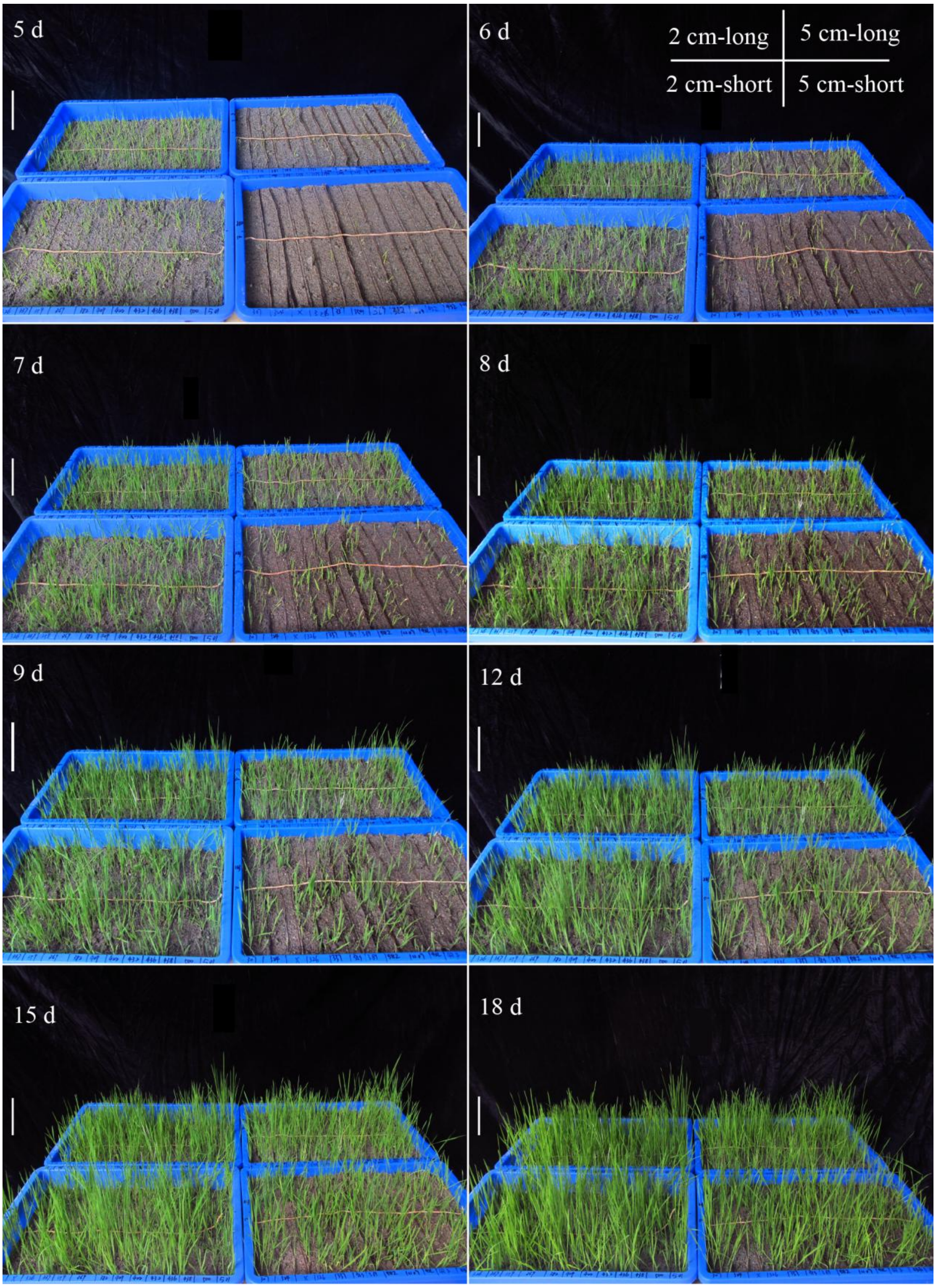

Figure S1 The direct-seeding emergence of accessions with long and short mesocotyl lengths at 2- and 5-cm sowing depths. Bar = 5 cm. The material distribution was shown in the upper right corner.
