# Supplementary figures and images for: Uncovering novel loci for mesocotyl elongation and shoot length in indica rice through genome-wide association mapping
Source: Planta. 2015 Nov 26;243:645–57. doi: 10.1007/s00425-015-2434-x (PMC4757631; doi:10.1007/s00425-015-2434-x)

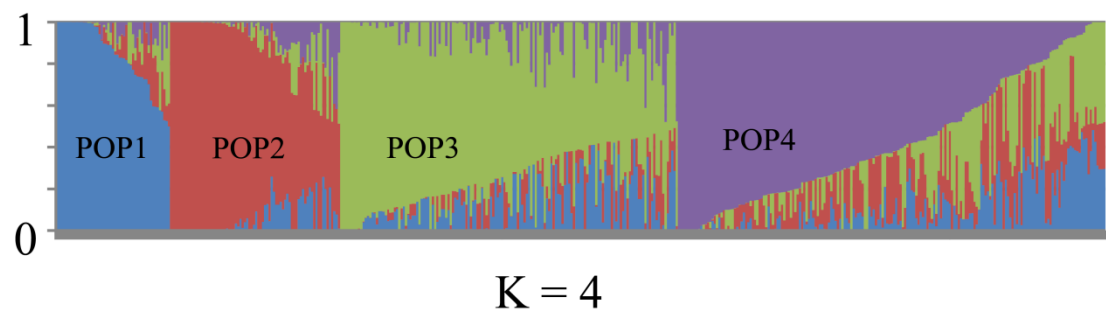

Figure S2 Model-based population assignment of 469 indica accessions.

Supplement: Supplementary file 2 — Supplementary material 2 (PDF 44 kb) [file 425_2015_2434_MOESM2_ESM.pdf]

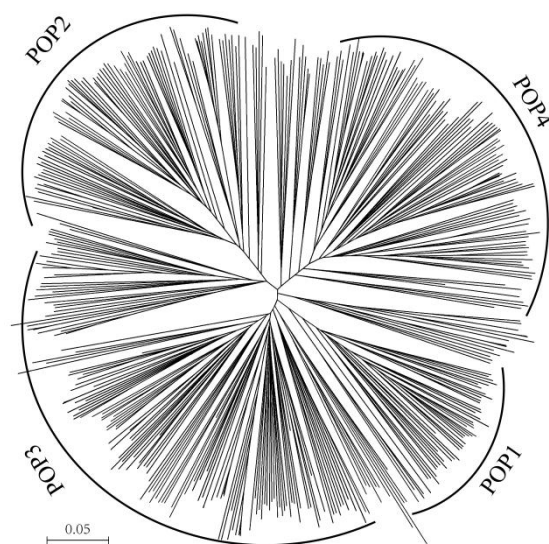

Figure S3 Neighbor-joining tree of 469 indica accessions.

Supplement: Supplementary file 3 — Supplementary material 3 (PDF 122 kb) [file 425_2015_2434_MOESM3_ESM.pdf]
