# Supplementary material for: Uncovering novel loci for mesocotyl elongation and shoot length in indica rice through genome-wide association mapping
Source: Planta. 2015 Nov 26;243:645–57. doi: 10.1007/s00425-015-2434-x (PMC4757631; doi:10.1007/s00425-015-2434-x)
Supplement: Supplementary file 4 — Supplementary material 4 (PDF 309 kb) [file 425_2015_2434_MOESM4_ESM.pdf]

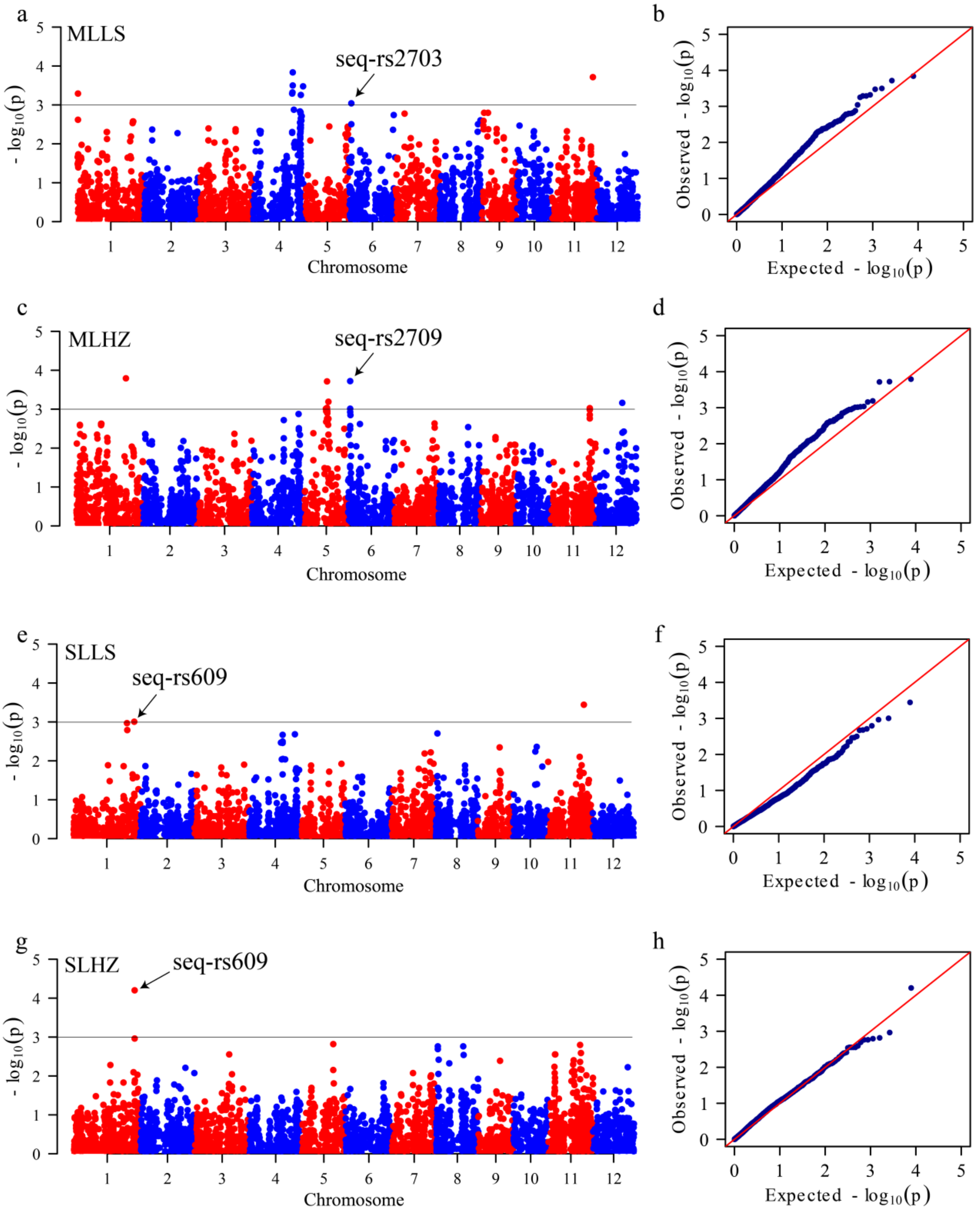

Figure S4 Manhattan and quantile-quantile plots for mesocotyl and shoot lengths in two environments. (a), (c), (e) and (g): Manhattan plot showing *P*-values along the genome calculated by compressed mixed linear model; (b), (d), (f) and (h): Q-Q plot showing the expected null distribution of *P*-values (expected *P*-values), assuming no association, represented as a red line and distribution of *P*-values (Observed *P*-values) represented as blue dots. MLLS, GWAS for mesocotyle length in Lingshui; MLHZ, GWAS for mesocotyl length in Hangzhou; SLLS, GWAS for shoot length in Lingshui; SLHZ, GWAS for shoot length in Hangzhou.
