# Supplementary material for: Uncovering novel loci for mesocotyl elongation and shoot length in indica rice through genome-wide association mapping
Source: Planta. 2015 Nov 26;243:645–57. doi: 10.1007/s00425-015-2434-x (PMC4757631; doi:10.1007/s00425-015-2434-x)
Supplement: Supplementary file 5 — Supplementary material 5 (PDF 75 kb) [file 425_2015_2434_MOESM5_ESM.pdf]

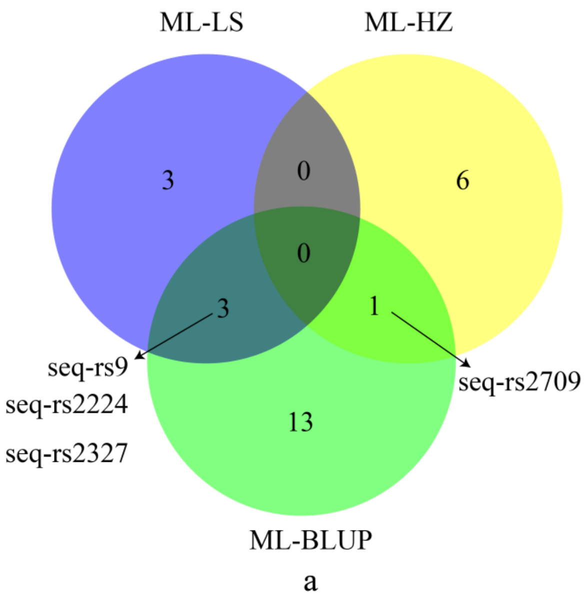

Figure S5 Venn plots of trait-marker associations for mesocotyl and shoot lengths. (a) Mesocotyl length; (b) Shoot length. ML, Mesocotyl length; SL, Shoot length; LS, Lingshui; HZ, Hangzhou; BLUP, Best linear unbiased prediction for phenotypic value in Lingshui and Hangzhou.
